# Postural Balance and Gait Parameters of Independent Older Adults: A Sex Difference Analysis

**DOI:** 10.3390/ijerph19074064

**Published:** 2022-03-29

**Authors:** Jessica Espinoza-Araneda, Valeria Bravo-Carrasco, Cristian Álvarez, Gabriel Nasri Marzuca-Nassr, Carmen Luz Muñoz-Mendoza, Javier Muñoz, Cristian Caparrós-Manosalva

**Affiliations:** 1 Department of Human Movement Sciences, Faculty of Health Sciences, University of Talca, Talca 3460000, Chile; jeespinoza@utalca.cl; 2 Interuniversity Center for Healthy Aging, Curico 3340000, Chile; cristian.alvarez@ulagos.cl (C.Á.); gabriel.marzuca@ufrontera.cl (G.N.M.-N.); calmunoz@ubiobio.cl (C.L.M.-M.); 3 Faculty of Engineering, University of Talca, Curico 3340000, Chile; vbravocarrasco@gmail.com (V.B.-C.); jamunoz@utalca.cl (J.M.); 4 Department of Health, University of Los Lagos, Osorno 5290000, Chile; 5 Exercise and Rehabilitation Sciences Laboratory, School of Physical Therapy, Faculty of Rehabilitation Sciences, Universidad Andres Bello, Santiago 7591538, Chile; 6 Department of Internal Medicine, Faculty of Medicine, Universidad de La Frontera, Temuco 4780000, Chile; 7 Nursing Department, Faculty of Health and Food Sciences, University of Bío-Bío, Chillan 3780000, Chile

**Keywords:** postural balance, gait analysis, aging, sex distribution

## Abstract

Postural balance and gait are important factors in the functional status of older people; however, few studies have addressed differences by sex. The objective of this study was to analyze the postural balance and temporal–spatial parameters of gait in independent older adults by sex. A cross-sectional study was conducted. Thirty-eight independent older women (69 ± 5 years), and 33 men (71 ± 5 years) were evaluated. The postural balance test with open and closed eyes was performed on two surfaces (hard/soft) on a force platform. Gait was recorded with cameras to analyze cycle duration and speed, step length, stride length, and foot clearance. The area of postural balance was greater in men in all tests (*p* < 0.001). Foot clearance height and cycle duration were lower in women (*p* < 0.05). Men showed a negative correlation between the area of balance and gait parameters. In women, a positive correlation was observed between foot clearance and balance with eyes closed. The postural balance and gait suggest discrepancies by sex, showing that older men behave differently according to the requirement of the motor task compared to women. These findings suggest being corroborated in more complex studies in the future.

## 1. Introduction

The rapid growth of the elderly population has implied an increase in the demands for prevention and care, giving particular importance to functional care [1]. In Chile, 85.8% of older people are independent, meaning that this cohort is capable of performing basic activities of daily life, without the need for help [2]. However, it is known that advancing age, together with unhealthy lifestyles, produces negative changes in the musculoskeletal structures, impacting the function of organs and systems. This is a major risk for the development of geriatric syndromes and functional limitations [3,4]. Falls, for example, are considered a highly prevalent geriatric syndrome, which can be present in different functional conditions and are associated with higher morbidity and mortality [5].

The main risk factors for suffering a fall in older people are associated with postural balance alterations and gait disturbances [4,6], and it has been discussed whether these factors may be influenced by sex. Preliminary studies have shown that the difficulties in balance tests seem to be greater in men than in women [7,8,9]. For example, older men have shown less precision and greater oscillation in the position of the center of mass, especially in conditions of somatosensory and visual deprivation, associated with changes in postural control strategies [10,11,12]. However, the literature still shows inconsistencies. Concerning the performance of standing postural balance tests, some studies report that older women show poorer performance in this variable [4,13]; although, on the contrary, other studies have observed poorer performance in men [8,14,15], while others find no differences by sex at all [16]. These discrepancies may be due to the type of test used and the population cohort studied. Balance can be assessed using either clinical balance tests—such as the Tinetti test, Berg Balance Scale, timed up-and-go, among others—or more objective measures such as posturography. However, clinical tests only assess visible balance deficits, and they may be less sensitive in the older adults who are active and independent [17].

Gait is affected by aging, favoring the occurrence of falls [18]. There is a relationship between advanced age and alterations in gait spatiotemporal outcomes—such as speed, stride length, cadence, and foot clearance, among others—in which it has been observed that aging could have different effects between the sexes [19,20]. For example, according to previous studies, older women present shorter steps and a higher cadence than men [19,21], suggesting that the deterioration of the physiological systems involved in gait may be compensated differently between older men and women. In this regard, it has been observed that cumulative impairments in multiple physiological systems can make older adults more vulnerable to slow gait and stride length [22,23]. Physiological factors—such as age, height, and physical activity level—appear to affect differently to healthy older men and women [23].

An appropriate postural balance and gait are important functional indicators in older people, but the available evidence is still insufficient and inconsistent to determine whether the deterioration of these indicators associated with aging is different between men and women. Most studies address sex differences by analyzing balance and gait parameters separately and do not establish relationships between them. Based on this, the purpose of this study was to analyze the postural balance and temporal–spatial parameters of gait in independent older adults distributed according to sex and to establish the level of association between the balance and gait parameters.

## 2. Materials and Methods

### 2.1. Study Design

A cross-sectional study was performed on older people that have been classified as independent according to the instrument recommended by the Ministry of Health of Chile, Functional Examination of the Elderly (EFAM, according to the abbreviation in Spanish, MINSAL, Santiago, Chile, 2015) [24]. A measurement of the postural balance and gait was performed on all participants in the Human Movement Analysis Laboratory of the University of Talca, Chile. The participants attended wearing comfortable clothing and were evaluated in the same way in all tests carried out. To ensure the overall integrity of the investigation, all tests were performed at the same time (03:00 to 06:00 in the afternoon), under the same environmental temperature conditions (22 °C), without visual or auditory stimuli that might interrupt the tests.

### 2.2. Participants

A total of 123 older adults of both sexes were recruited from the city of Talca, Chile. This study followed the guidelines of the Declaration of Helsinki of the World Medical Association (2008) and was approved by the Scientific Ethics Committee of the University of Talca (ID 1A-2018). Using the software G*Power version 3.1.9.7 we calculated the sample size. Thus establishing an alpha error of *p* < 0.05 with a moderate effect size (ES) of 0.6, and a critical *t* value of 1.667, a sample of two independent groups for post hoc comparisons and correlational analyses requires a minimum of 71 subjects to give us a statistical power of 80%. The purpose and characteristics of the study were explained to all participants. Participants who voluntarily signed the informed consent were selected according to the following inclusion criteria: between 60 and 80 years of age, classified as independent using the EFAM instrument, presenting independent gait without assistance, and with any chronic diseases controlled. Participants who showed pathologies that affect balance or of neurological origin, uncorrected visual impairment, or history of frequent falls (presence of two or more falls in one year) were excluded. Finally, 71 older adults were selected (38 women and 33 men).

### 2.3. Procedure

Body weight and height were measured in all participants at the beginning of the session with a scale and calibrated stadiometer (Detecto^®^, Webb City, MO, USA). The body mass index (kg/m^2^) was calculated. Assessments of postural balance and gait were performed in a single session. The balance test was performed in a standing position on a force platform with the arms at the side, barefoot, relaxed, with eyes open (EO), with the sight in front on a reference mark located at the level of the eyes. The participant was asked to stay in that position for 30 s. Then, in the same position and posture but with eyes closed (EC), the position was held for another 30 s. These two methods of evaluation (EO and EC) were carried out on a hard surface (HS: the rigid surface of a platform) and a soft surface (SS: the surface of a medium density foam). Before the evaluation, the participants were familiarized with the tests and instruments and then recorded once with a rest interval of 1 min between each stage. The older adults were assisted at all times by the evaluators if required.

For the balance test, the center of pressure (CoP) was recorded using a force platform measuring 50 × 43 cm^2^ (Balance Plate BertecTM BP5050, USA). It was located 1.5 m away from the wall and the capture record was performed at 100 Hz with Digital Acquire V4 software (Bertec, Columbus, OH, USA). The support position of the feet was marked so it could be reproduced in all stages of the test. The records of the CoP obtained in the balance tests were registered and analyzed using MATLAB^®^ V15a software (The MathWorks, Inc., Natick, MA, USA). The area was determined from the ellipse of 95% of the data dispersion of the two axes (CoPx and CoPy) for each stage of the test [25].

To assess gait, each participant was instructed to walk barefoot in a 9-m well-lit corridor without any obstacles. Participants were instructed to walk at a comfortable, self-determined speed. The gait measurement was repeated three times. For gait measurement, a motion capture system with eight Optitrack infrared cameras (Naturalpoint, Inc., Corvallis, OR, USA) was used. A capture volume of 2 × 2 × 2 m^3^ was set to the center of the corridor and a capture rate of 100 fps. Sixteen reflective markers were installed on both sides of the lower limbs to create a conventional lower limb model.

The kinematic data obtained during walking was smoothed using a fourth order Butterworth low pass filter at 8 Hz and analyzed with Igor Pro V6.12 software (Wavemetrics, Portland, OR, USA). Each gait cycle was determined by visually detecting the heel contact frame up to the same foot contact. The variables of cycle duration (s), stride length (m), step length (m), and gait cycle speed (m/s) were obtained. The foot clearance was determined from the trajectory of the second toe marker to the ground and identified at the moment of lowest height during the swing phase [26]. Each gait variable was averaged over the three repetitions performed for later analysis. Because the mean heights of the groups were different, the values of stride length and step length were adjusted to the height of each participant (length/height × 100).

### 2.4. Statistical Analysis

For statistical analysis, the normality of all data was tested with the Shapiro–Wilk test (*p* > 0.05). Comparisons between age, height, weight, BMI, and EFAM (i.e., baseline outcomes), as well as the main comparisons between older men and women, were made using the multivariable ANOVA test, with the Games–Howell post hoc test. Subsequently, bivariate correlations between the postural balance and gait variables were calculated using Spearman’s rho test (***r_s_***). The size of the correlation coefficient was estimated considering the proportion or magnitude of ***r_s_***. A ratio ***r_s_*** = 0.1 indicated a small effect size, ***r_s_*** = 0.3 a medium effect size, and ***r_s_*** = 0.5 a large effect size. Additionally, for the analysis of differences by sex, the effect size partial eta squared (η^2^) was calculated [27] as additional information. To interpret η^2^, the recommended conventions were used [27], where small (η^2^ = 0.01), medium (η^2^ = 0.06), and large (η^2^ = 0.14) effects. All analyses were performed using SPSS V28 (SPSS Inc., Chicago, IL, USA) software and statistical significance was established at *p* < 0.05.

## 3. Results

From the sample of 71 older adults, 38 were women (53.5%) and 33 were men (46.5%). The variables of age, weight, height, and BMI are presented in Table 1. The area of the CoP was different between the sexes in the tests carried out on both hard and soft surfaces and in both conditions of EO and EC (*p* ≤ 0.001, *p* = 0.004, respectively), showing an area of greater oscillation in men than women, with a large effect size for all parameters (Table 2).

Gait parameters (Table 2) such as foot clearance height and cycle duration showed significantly higher values in men compared to women, with a large effect size (*p* = 0.005, η^2^ = 0.991; *p* ≤ 0.001, η^2^ = 0.989, respectively). Cycle speed, step length, and stride length did not show differences between the sexes (*p* > 0.05).

**Figure 1:**
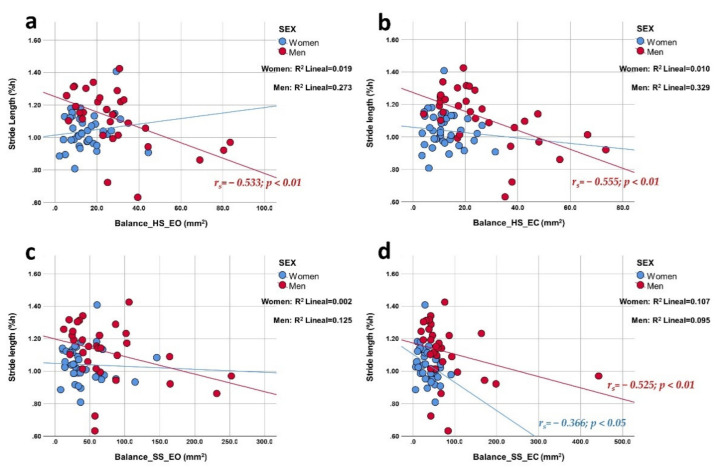
Correlation plots of stride length compared to balance area in each of the tests. Each image shows the correlations obtained distributed by sex. The upper part of the figure shows the results for a hard surface with eyes open (panel (**a**); linear R^2^ women: 1.9%; men: 27.3%) and closed eyes (panel (**b**); linear R^2^ women: 1.0%; men: 32.9%); the lower part shows the results for a soft surface with open eyes (panel (**c**); linear R^2^ women: 0.2%; men: 12.5%) and closed eyes (panel (**d**); linear R^2^ women: 10.7%; men: 9.5%).

**Figure 2:**
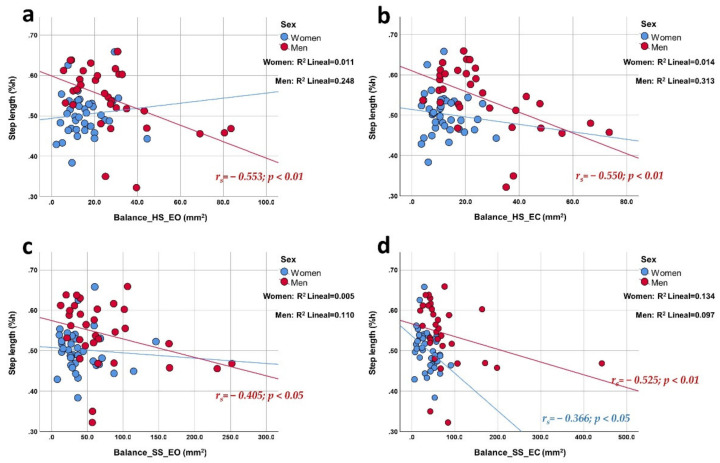
Correlation plots of step length compared to balance area in each of the tests. Each image shows the correlations obtained distributed by sex. The upper part of the figure shows the results for a hard surface with eyes open (panel (**a**); linear R^2^ women: 1.1%; men: 24.8%) and closed eyes (panel (**b**); linear R^2^ women: 1.4%; men: 31.3%); the lower part shows the results for a soft surface with open eyes (panel (**c**); linear R^2^ women: 0.5%; men: 11.0%) and closed eyes (panel (**d**); linear R^2^ women: 13.4%; men: 9.7%).

The Spearman rho test was performed to assess the correlation between the gait and balance parameters (Table 3). In older men, stride length (Figure 1) and step length (Figure 2) showed high and negative correlations in almost all balance tests, except in soft surface EO, which showed a moderate correlation. Women only presented a mean negative correlation in the soft surface EC test concerning both indicated gait parameters.

**Figure 3:**
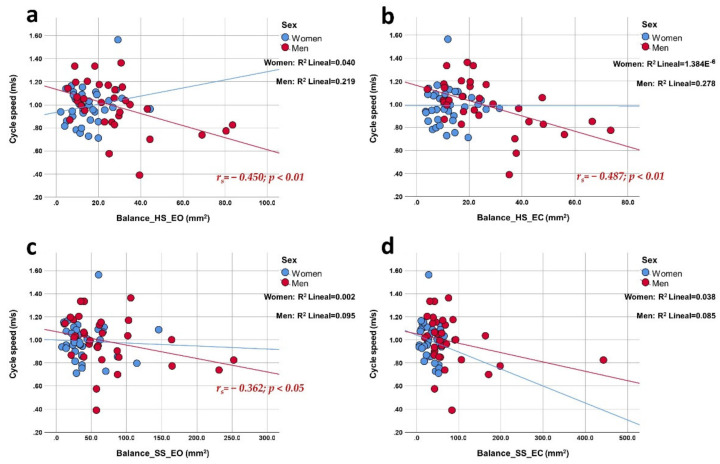
Correlation plots of gait cycle speed compared to balance area in each of the tests. Each image shows the correlations obtained distributed by sex. The upper part of the figure shows the results for a hard surface with eyes open (panel (**a**); linear R^2^ women: 4.0%; men: 21.9%) and closed eyes (panel (**b**); linear R^2^ women: 0.0%; men: 27.8%); the lower part shows the results for a soft surface with open eyes (panel (**c**); linear R^2^ women: 0.2%; men: 9.5%) and closed eyes (panel (**d**); linear R^2^ women: 3.8%; men: 8.5%).

**Figure 4:**
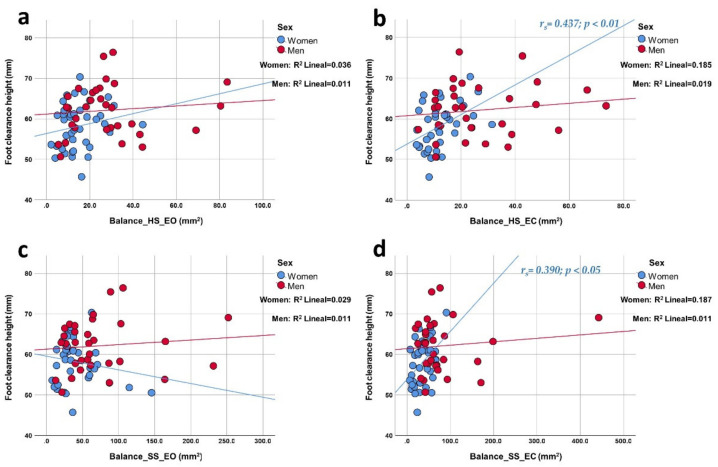
Correlation plots of foot clearance height compared to balance area in each of the tests. Each image shows the correlations obtained distributed by sex. The upper part of the figure shows the results for a hard surface with eyes open (panel (**a**); linear R^2^ women: 3.6%; men: 1.1%) and closed eyes (panel (**b**); linear R^2^ women: 18.5%; men: 1.9%); the lower part shows the results for a soft surface with open eyes (panel (**c**); linear R^2^ women: 2.9%; men: 1.1%) and closed eyes (panel (**d**); linear R^2^ women: 18.7%; men: 1.1%).

In men, the speed of the gait cycle (Figure 3) presented a medium negative correlation with balance in all tests except EO on a soft surface. Women, on the other hand, did not show correlations for this gait parameter. The height of the foot clearance (Figure 4) presented a medium positive correlation in women in the EC tests on both surfaces. Men did not show correlations for this gait parameter. The time of the gait cycle did not show correlations with the area of the CoP by sex. Additionally, the correlation graphs (Figure 1, Figure 2 and Figure 3) give us information about a higher predictive value observed for both stride length, step length, and gait cycle speed on postural balance in HS (>27.3%, >25%, and >22%, respectively) in older men compared to older women. On the other hand, foot clearance height (Figure 4) showed a higher predictive value in older women, mainly in the tests with eyes closed (~18%).

## 4. Discussion

The purpose of this study was to analyze the postural balance and temporal-spatial parameters of gait in independent older adults distributed by sex. The main results obtained show that men present a greater CoP displacement area in the static balance test in all conditions compared to women (HS-EO; HS-EC; SS-EO; SS-EC). Regarding gait parameters, the cycle speed, stride length, and step length did not show significant differences between men and women. Foot clearance height and gait cycle duration were significantly greater in men. Negative correlations were observed in men between the CoP oscillation area in all sensory conditions concerning stride length, step length, and gait cycle speed. The women showed significant negative correlations between balance evaluated on a soft surface with eyes closed and the length of the stride and step; and interestingly, a positive correlation with the foot clearance height.

The increase in the number of older people is associated with greater functional dependence in society, and alterations in postural balance and gait contribute significantly to this greater dependence [28]. It is known that aging generates deleterious changes in postural control systems [9,17], affecting the adequate control of CoP displacement, generating reduced postural stability and increased risk of falling [17,29]. However, the differences in standing postural balance and gait according to sex are still poorly addressed in the literature. According to Riva et al. [13], women over 75 years of age are less stable than men in one-legged stance balance. Kim et al. [4], on the other hand, reported that women presented greater age-related medial-lateral CoP displacement compared to men, but not in the anteroposterior direction. Our results agree with previous studies that have shown poorer CoP parameters in older men compared to women, mainly with the manipulation of the sensory system (closed eyes or soft surface), presumably explained by a greater somatosensory and proprioceptive decline in men [9,15,30].

Studies have reported that the greater postural sway in men may be due to anthropometric characteristics such as greater height, which raises the center of mass (CoM), increasing the demand for postural control strategies compared to women [9,14]. From pubertal age, men tend to be taller than women; however, no differences in CoP shift have been found between men and women in the pre-aging stages [31]. In our study, older men show significantly higher height and weight than women, which could be a factor that influences the performance of the balance tests. Thus, it could be speculated that men achieve efficient balance control mechanisms at younger ages, but with age, it would be more difficult to control postural balance due to decreased sensory inputs from the visual or somatosensory systems, generating a greater dependence on these systems compared to women. In this sense, it would be advisable to incorporate the analysis of the height of the CoM in balance tests distributed by sex in future studies.

The correlations obtained between the area of postural balance and gait parameters are interesting. In older men, poorer performance in balance tests was correlated with shorter stride length, step length, and gait cycle speed, whereas only the most demanding test (SS-EC) showed this correlation for both sexes. Few studies have addressed the correlation between these functional indicators in older adults distributed by sex. Puszczalowska-Lizis et al. [9] mentioned that these differences may be due to the different postural strategies adopted by men and women in response to the deterioration of the postural control mechanisms associated with aging, with older men being more susceptible to this condition. The negative correlation observed between the more complex balance test and stride length and step length, observed in both sexes, could be part of a cautious attitude when walking as a strategy to maintain task stability [32,33].

The foot clearance height has been reported as a critical control factor of the oscillation phase associated with trips and falls [34,35]. Around 53% of falls are due to tripping during walking [34], related to the difficulties for an adequate trajectory of the swinging foot when facing some unexpected obstacle [36]. The reduction in the foot clearance height for a given step during walking increases the susceptibility to tripping. Our results showed a significantly lower foot clearance height in women, which would indicate a higher risk of tripping. This is consistent with a previous study that have shown that older women with lower foot clearance fall more frequently compared to older men [37]. Among the causes, it has been reported that older women seem to have less ankle dorsiflexion and generate greater hip flexion as a compensatory mechanism to avoid falling [35].

Few studies correlate balance with the foot clearance height. Our results show that older women who perform worse on the more complex balance tests demonstrate a higher foot clearance height compared to men. Ribeiro et al. [35], point out that when studying the foot clearance height in older women compared to young women, the observed decrease could be influenced by the motor deterioration typical of aging, as well as by factors such as the decrease in muscle strength, flexibility, and postural control in older adults. Although it seems that the greater height observed in the older women in our study could respond to a compensatory strategy when they present a poorer performance in balance tests with somatosensory disturbance (SS), it is necessary to understand more about the motor control mechanisms involved in the oscillation phase and its relationship with factors such as muscle strength and proprioception in older people of different sexes.

A limitation of this study is the sample size of each group and that it did not include adults over 80 years of age. Although the scope of the correlations may be limited, few studies propose sex-differentiated analyses between balance and gait parameters, and we believe that future studies should attempt these differentiated analyses. On the other hand, not including the comparison with dependent older adults, the level of physical activity—as well as the drugs used by each participant—limits the discussion of the control factors of posture and gait that can explain the differences identified by sex. It is necessary for other investigations that include older age groups to observe the impact of functional dependence, as well as the risk of falling, on these variables. The CoP area of postural balance and the temporal-spatial parameters of gait are variables recognized and used by studies in older adults; future research should consider not only static balance measures. Dynamic and reactive balance measures can provide novel and interesting knowledge in the differentiation by sex in older people. Finally, the study does not allow establishing the causes of the differences by sex in the variables analyzed. Future studies should incorporate the assessment of possible causal factors, such as neuromuscular, sensory, and proprioceptive parameters when analyzing the differences by sex in these cohorts.

## 5. Conclusions

Sex differences in postural balance and gait are important in describing performance skills in older adults. Our results suggest discrepancies in test performance between older men and women. Older men show less postural balance control compared to older women. In addition, older men show a negative correlation between postural balance control with gait parameters, except for foot clearance. Older men and women would behave differently depending on the requirements of the motor task. These findings and the mechanisms that underlie these differences suggest being corroborated in more complex studies in the future.

## Figures and Tables

**Table 1:** Characteristics of the participants are distributed by sex. Data are presented as mean (standard deviation).

Characteristics	Women (*n* = 38)	Men (*n* = 33)	*p*-Value *
Age (years)	69 (5)	71 (5)	0.208
Height (m)	1.52 (0.05)	1.68 (0.06)	**<0.001**
Body Weight (kg)	68.7 (10.3)	81.6 (10.2)	**<0.001**
BMI (kg·m^−2^)	29.3 (3.8)	28.7 (3.6)	0.486
EFAM (score) **	50.5 (3.1)	51.7 (2.0)	0.058

* *p*-values in bold represent significant differences. ** The dependency classification score according to EFAM is <42.

**Table 2:** Comparison by sex of the postural balance and gait variables. Data are presented as Median (interquartile range).

Outcome		Women (*n* = 38)	Men (*n* = 33)		
CoP area (mm^2^)		M(IR)	M(IR)	*p*-value	η^2^
Hard surface	EO	12.7 (10.4)	25.1 (19.6)	**<0.001**	**0.683**
	EC	11.1 (7.2)	20.4 (26.0)	**<0.000**	**0.696**
Soft surface	EO	31.7 (33.8)	57.2 (51.9)	**0.004**	**0.621**
	EC	34.8 (35.1)	56.0 (39.6)	**0.004**	**0.522**
Gait		M(IR)	M(IR)	*p*-value	η^2^
Foot clearance height (mm)		58.7 (8.2)	62.8 (9.2)	**0.005**	**0.991**
Cycle duration (s)		1.04 (0.12)	1.15 (0.14)	**<0.001**	**0.989**
Cycle speed (m/s)		0.99 (0.19)	1.00 (0.30)	0.944	0.967
Stride length (% height)		0.67 (0.08)	0.69 (0.11)	0.473	0.987
Step length (% height)		0.32 (0.04)	0.33 (0.06)	0.618	0.988

CoP: Center of Pressure; EO: Eyes-open; EC: Eyes-closed; s: seconds; m: meters; mm: millimeters; m/s: meters/seconds; η^2^: effect size partial eta squared. *p*-values in bold represent significant differences.

**Table 3:** Correlation between postural balance and gait parameters in the older population of the present study.

Correlation	Hard Surface				Soft Surface			
	Balance EO (mm^2^)		Balance EC (mm^2^)		Balance EO (mm^2^)		Balance EC (mm^2^)	
Cycle duration (s)	***r_s_***	***p***	***r_s_***	***p***	***r_s_***	***p***	***r_s_***	***p***
Men	0.149	0.405	0.220	0.218	0.102	0.571	0.087	0.629
Women	0.054	0.746	−0.040	0.810	0.107	0.519	−0.015	0.926
Cycle speed (m/s)								
Men	−0.450 **	**0.008**	−0.487 **	**0.004**	−0.362 *	**0.038**	−0.341	0.052
Women	0.039	0.811	0.027	0.871	−0.190	0.251	−0.203	0.220
Foot clearance height (mm)								
Men	0.078	0.662	0.080	0.657	0.165	0.358	0.025	0.887
Women	0.242	0.141	0.437 **	**0.006**	0.037	0.824	0.390 *	**0.015**
Stride length (%height)								
Men	−0.521 **	**0.002**	−0.483 **	**0.004**	−0.486 **	**0.004**	−0.398 *	**0.022**
Women	0.094	0.575	−0.098	0.559	−0.191	0.251	−0.345 *	**0.034**
Step length (%height)								
Men	−0.544 **	**0.001**	−0.397 *	**0.022**	−0.434 *	**0.012**	−0.393 *	**0.024**
Women	0.124	0.460	−0.122	0.466	−0.188	0.259	−0.365 *	**0.024**

EO: Eyes-open; EC: Eyes-closed; s: seconds; m: meters; mm: millimeters; m/s: meters/seconds; ***r_s_***: Spearman’s rho; *p*-values in bold represent significant differences. * *p* < 0.05; ** *p* < 0.01.

## Data Availability

Data from this study is available in the following http://doi/10.6084/m9.figshare.19178732 (accessed on 15 February 2022).
